# Perfluorocarbon-Based Oxygen Carriers and Subnormothermic Lung Machine Perfusion Decrease Production of Pro-Inflammatory Mediators

**DOI:** 10.3390/cells10092249

**Published:** 2021-08-30

**Authors:** Stephan Arni, Citak Necati, Tatsuo Maeyashiki, Isabelle Opitz, Ilhan Inci

**Affiliations:** Department of Thoracic Surgery, University Hospital Zürich, 8091 Zürich, Switzerland; stephan.arni@usz.ch (S.A.); necomomus@gmail.com (C.N.); tmaeya@juntendo.ac.jp (T.M.); Isabelle.Schmitt-Opitz@usz.ch (I.O.)

**Keywords:** ex vivo lung perfusion, subnormothermic perfusion, perfluorocarbon-based oxygen carriers, lung transplantation

## Abstract

The quality of marginal donor lungs is clinically assessed with normothermic machine perfusion. Although subnormothermic temperature and perfluorocarbon-based oxygen carriers (PFCOC) have proven favourable for other organ transplants, their beneficial use for ex vivo lung perfusion (EVLP) still requires further investigation. In a rat model, we evaluated on a 4 h EVLP time the effects of PFCOC with either 28 °C or 37 °C perfusion temperatures. During EVLP at 28 °C with PFCOC, we recorded significantly lower lung pulmonary vascular resistance (PVR), higher dynamic compliance (Cdyn), significantly lower potassium and lactate levels, higher lung tissue ATP content, and significantly lower myeloperoxidase tissue activity when compared to the 37 °C EVLP with PFCOC. In the subnormothermic EVLP with or without PFCOC, the pro-inflammatory mediator TNFα, the cytokines IL-6 and IL-7, the chemokines MIP-3α, MIP-1α, MCP-1, GRO/KC as well as GM-CSF, G-CSF and the anti-inflammatory cytokines IL-4 and IL-10 were significantly lower. The 28 °C EVLP improved both Cdyn and PVR and decreased pro-inflammatory cytokines and pCO2 levels compared to the 37 °C EVLP. In addition, the 28 °C EVLP with PFCOC produced a significantly lower level of myeloperoxidase activity in lung tissue. Subnormothermic EVLP with PFCOC significantly improves lung donor physiology and ameliorates lung tissue biochemical and inflammatory parameters.

## 1. Introduction

To date, lung transplantation is an accepted modality of care for patients with end-stage lung diseases. Nonetheless, severe case of allograft ischemia-reperfusion (I/R) injury leads to primary graft dysfunction and remains a significant cause of early morbidity and mortality after lung transplantation [1]. The shortage of healthy lungs for transplantation demands optimal utilization of the available donor pool whereas advances in organ preservation and donor management have all sought to increase the rate of organ usage from the current donor pool [2]. Novel strategies have been implemented to overcome this shortage, such as application of marginal donor lungs [3], donation after cardiac death donors, living donor lobar lung transplantation, and ex vivo lung perfusion (EVLP). EVLP is a technique that continues to evolve and influence clinical practice [4] and allow to re-evaluate questionable donor lungs for assessment [5], and resuscitation and repair of extended criteria donor lungs [6]. Normothermic EVLP for highly metabolically active organs requires that the physiological environment is restored with full nutritional support. The preservation of the transplantable and highly metabolic abdominal grafts, the liver and the kidneys, has been improved by mimicking physiological conditions and allowing the continuous elimination of toxic products from the cellular medium while carrying out an active restoration of ATP reserves and glycogen [7,8]. Signs of oxidative and inflammatory stress have been monitored in liver perfusate with metabolomics and principal component analysis approaches [9]. Researchers have also suggested how the initial phase of hypothermic oxygen infusion soon after graft removal promoted the reduction of wastes, such as succinates, which typically accumulate in the graft during static storage, and have developed protocols that have allowed the restoration of mitochondrial ATP levels, reduced NADH/NAD ratio, a decrease in lactate, succinate and purine derivatives [7,10]. Although cold storage at 4 °C reduces the lung tissue metabolic rate, the metabolism is not fully put to a complete rest due to the decreased metabolic rate [11]. The main reasons behind performing lung subnormothermic or hypothermic machine perfusions are to mitigate damages due to I/R injury [12], to decrease production of reactive oxygen species (ROS), and to reduce carbon dioxide production and, consequently, decrease the requirement for minute ventilation. Moreover, the use before transplantation of hypothermic oxygenated machine perfusion has been reported to preserve kidneys and livers with better results than cold static storage [13]. Oxygenated ex vivo perfusions were successfully tested at 22 °C in porcine kidneys with hemoglobin-based oxygen carriers [14] and at 4 °C in donor rat livers with perfluorocarbon-based oxygen carriers (PFCOC) [15]. PFCOC have been developed to mimic blood oxygen transport properties and have relatively short half-life (12–24 h) but ex vivo organ machine perfusion studies showing clear benefits for the use of PFCOC are still scarce [16]. PFCOC are chemically and biologically inert and can dissolve large amounts of oxygen, carbon dioxide, and other gases [17,18]. As perfluorocarbon are immiscible in water they have to be emulsified for intravascular administration in human clinical trials [19]. PFCOC are emulsions taking advantage of the high solubility of respiratory gases in perfluorocarbons [19]. Due to the small size (0.1–0.2 microns versus 7–8 microns), PFCOC emulsion particles mainly flow in the peripheral plasma layer in larger vessels. PFCOC perfuses even the tiniest capillaries, where no red blood cells may flow under certain conditions (i.e., micro blood clots) in the microcirculation. With PFCOC, there is no saturation and no possibility for chemical binding and interferences with carbon monoxide, nitric oxide and other reagents. With PFCOC present in the lung microcapillaries, the oxygen is released and the carbon dioxide is picked up. In addition, the systemic administration of PFCOC may have potent anti-inflammatory effects in vivo [19,20,21] ex vivo [22] and in vitro [23,24].

The real-time monitoring of lung graft procurement physiology and mechanics have not yet been studied with the use of PFCOC-containing perfusates at subnormothermic temperature. We document the cytoprotective benefits of reduced cellular metabolism and the protective effect against pro-inflammatory tissue mediators with the use of subnormothermic lung perfusion and PFCOC as a non-inferior setting in comparison to the clinically approved normothermic lung perfusion.

## 2. Materials and Methods

### 2.1. Animals

The Veterinarian committee Kanton Zurich approved the animal use for this study (ZH160/18). The male Sprague Dawley rats (Janvier Labs, Le Genest Saint-Isle, France) weighing 280 to 360 g were maintained in a pathogen-free environment and received adequate care according to “Guide for the Care and Use of Laboratory Animals: Eighth Edition” [25]. This study was carried out in compliance with the ARRIVE guidelines.

### 2.2. Rat Donor Lung Procurment Techniques

All rats were anaesthetized with 2–3% (*v*/*v*) isoflurane in O_2_. After fully anesthetized the animal were orotracheally intubated with a 14-gauge intravenous catheter and underwent mechanical ventilation with a rodent ventilator (Harvard Apparatus, Inc., Model Ventelite, Holliston, MA, USA). Rats were applied a volume control ventilation at a respiratory rate of 60 breaths/min with a tidal volume of 10 mL/kg, an inspired oxygen fraction (FiO_2_) of 1 and a positive end-expiratory pressure (PEEP) set at 3 cmH_2_O. After laparotomy, the animals were heparinized with 300 IU intravenous sodium heparin via the inferior vena cava. Thereafter, a median sternotomy was performed. On the anterior surface of the right ventricular outflow tract, we performed a 2–3 mm incision and placed the cannula inside this incision and into the main pulmonary artery and finally secured the cannula with a silk suture. Next, on the apex of the left ventricle, we made a 4–5 mm incision, placed a cannula into this incision and pushed it through the mitral valve into the left atrium. Following cannulation, the inferior vena cava was opened and we performed an anterograde flush with 20 mL Perfadex plus^®^ (XVIVO Perfusion, Uppsala, Sweden) at a perfusion pressure of 20 cmH_2_O. The lungs were inflated with a sustained airway pressure of 15 cmH_2_O and the trachea was clamped. We placed the harvested heart-lung block in a plastic bag containing 15 mL ice cold Perfadex plus^®^ for a 1 h time of cold ischemic time.

### 2.3. EVLP Procedure and Physiological Variables

An Isolated Perfused Lung System for rat and guinea pig (IPL-2, Hugo Sachs Elektronik Harvard Apparatus, March-Hugstetten, Germany) was used for EVLP under positive pressure ventilation. As an acellular perfusate, we selected 125 mL of Steen solution (Steen solution, XVIVO Perfusion AB, Göteborg, Sweden) supplemented with 300 IU sodium heparin, antibiotic (50 mg meropenem, Labatech Pharma, Meyrin, Switzerland), and methylprednisolone (50 mg Solu-Medrol, Pfizer Inc., New York, NY, USA). Prior to organ perfusion, we equilibrated the IPL-2 circuit perfusate for dissolved oxygen content into the 125 mL perfusate either without or with 0.2 gr PFCOC emulsion (0.1 gr PFCOC/kg body weight). The equilibrium of dissolved O_2_ content into the IPL-2 circuit perfusate was reached at 20 °C after 15 min at a 30 mL/min flow through a gas exchange membrane (D-150 hemofilter, Medsulfone Medica S.p.A., Medolla, Italy) with 2 L per minute flow of 100% oxygen. The 100% targeted flow was calculated as the 20% of a 250 g weight rat with a 75 mL/min cardiac output. We started lung perfusion with 10% of the targeted flow (1.5 mL/min) for 10 min. The five following 10 min steps in ml/min were 3 mL/min, 4.5 mL/min, 7.5 mL/min, 12 mL/min. Then at the 50 min time point we switched to maximum flow of 15 mL/min. The circuit perfusate temperature was gradually increased using a thermostatic water bath and the targeted temperatures of 37 °C or 28 °C were reached after 20 min or 10 min respectively and kept onwards during the 4 h time EVLP. The left atrium pressure was set at 2–3 cmH_2_O and the automatized IPL-2 controller system maintained the pulmonary arterial pressure (PAP) below 15 cmH_2_O by adjusting the flow. Ventilation with the IPL-2 ventilator (VCM-P, Hugo Sachs Elektronik Harvard Apparatus, March-Hugstetten, Germany) started at 28 °C or 37 °C perfusate temperature after 20 min reperfusion time and with 30% of the targeted flow. The fixed tidal volume was at 5 mL/kg, with an inspiratory/expiratory ratio of 1/3 and a rate of 30 breaths/min, a PEEP of 3 cmH_2_O and an inspired oxygen fraction (FiO_2_) of 0.21. Thereafter, the perfusate was deoxygenated with a mixture of 8% CO_2_ and 92% N_2_ using a gas exchange membrane (D-150 hemofilter, Medsulfone Medica S.p.A., Medolla, Italy). We recorded all the respiratory parameters with a dedicated software (PULMODYN^®^ HSE, Hugo Sachs Elektronik Harvard Apparatus, March-Hugstetten, Germany). We monitored PAP, peak airway pressure and airway flow during the 4 h time of EVLP. Hourly, and 5 min after switching ventilation with an inspired oxygen fraction FiO_2_ of 1, we recorded the dynamic lung compliance (Cdyn), the pulmonary vascular resistance (PVR) and sampled the perfusate. At the end of the 4 h of EVLP, we performed an additional stress test where the IPL-2 controller system was disabled allowing the flow to increase over the 100% targeted flow up to the maximum PAP value set at 15 cmH_2_O. After 5 min of stress test, we recorded flow, PVR and Cdyn. Before and after EVLP evaluation the heart lung block were weighted. For further analysis an aliquot of the lungs was flash frozen in liquid nitrogen. All samples were stored at −80 °C until further examinations. The number of EVLP performed, with or without PFCOC, was *n* = 6 for the subnormothermic groups and *n* = 6 for the normothermic groups.

### 2.4. Clinical Biochemistry Parameters

We used the Epoc^®^ blood analysis system (Epoc^®^ Blood Analysis System, Siemens Healthineers, Erlangen, Germany) for pH, concentrations of calcium, potassium, glucose, sodium, lactate and the partial oxygen pressure. The change in pO_2_ (ΔpO_2_) was calculated according to the following equations: ΔpO_2_ = partial pulmonary venous pO_2_ − pulmonary arterial pO_2_).

### 2.5. PFCOC Emulsion Preparation

Perfluorooctyl bromide (PFOB) (ABCR, Karlsruhe, Germany) was prepared by high-pressure homogenization [26]. The formulation of the PFOB emulsion consisted of 70% *w*/*v* PFC and 4% *w*/*v* egg yolk phospholipids (EYP, Lipoid E 80; Lipoid GmbH) in a phosphate buffer (0.052 NaH_2_PO_4_ · H_2_O, 0.355 Na_2_HPO_4_ · 7H_2_O, 0.25 NaCl; all in % *w*/*v*). Emulsions were stabilized by adding a semifluorinated alkane, mixed fluorocarbon/hydrocarbon diblock compound (C_6_F_13_C_10_H_21_, F_6_H_10_; equimolar with EYP). Emulsification was achieved under high pressure (1000 bar, 30  min) with a laboratory processor (Microfluidizer M-110, Microfluidics Corp., Newton, MA, USA). The emulsion was then heat sterilized (121  °C, 15 psi, 15  min). The particle size and zeta potential of the different emulsions were measured by dynamic light scattering using a Zetatrac™ (Particle Metrix, Meerbusch, Germany).

### 2.6. Cytokines, Chemokines and Mediators of Wound Healing and Tissue Repair

The perfusates collected after 4 h of EVLP were flash frozen in liquid nitrogen and stored at −80 °C. We assayed 50 µL of perfusate for cytokines, chemokines and mediators of wound healing and tissue repair levels with a mouse cytokine/chemokine panel (Bio-Plex Pro Mouse Cytokine 23-plex, Bio-Rad Laboratories, Hercules, CA, USA) according to the manufacturer’s instructions.

### 2.7. Estimates of ATP Content and Myeoloperoxidase Activity in Lung Tissue

Frozen lung tissue (25 mg) was powdered on dry ice and homogenized in 0.5 mL of 0.5% trichloroacetic acid. We centrifuged the lysates for 2 min at 4 °C and 8000 rpm to separate cleared supernatant from insoluble cell debris. The sample supernatants were buffered with 10 × concentrated Tris-acetate buffer containing 10 µL of 0.002% xylenol blue as pH indicator. Furthermore, sample pellets were reconstituted to their original volume with 1 × PBS and used to determine the protein concentrations with the Pierce microBCA protein assay kit (Thermo Scientific, Rockford, IL, USA) according to the manufacturer’s instructions and bovine serum albumin as standard. We used an ATP assay kit (Enliten, Promega, Madison, WI, USA) to estimate the ATP concentration in the supernatant by measuring in the luminescence channel of a Cytation 5 plate reader (BioTek Instruments, Inc., Winooski, VT, USA). The results are expressed in nanomolar ATP per milligram of proteins. Tissue lysate extracted from the powdered lung tissue was also analysed using a myeloperoxidase (MPO) activity assay (OxiSelect™ myeloperoxidase chlorination activity assay, Cell Biolabs San Diego, CA, USA) and according to manufacturer’s instructions. The results are expressed in milliunits per milligram of proteins.

### 2.8. Statistical Method

Results are presented as mean and standard deviation (SD). The median and interquartile range (IQR) were used for cytokine analysis as measures of central tendency and dispersion, respectively. A nonparametric Mann–Whitney U-test was used for non-continuous data. Three-way analysis of variance (ANOVA) and Tukey test (alpha = 0.05) was performed to investigate the main effects of the independent variables and the interactive effects between them. Statistical analysis was performed with GraphPad Prism version 8 software (GraphPad Software, Inc., La Jolla, CA, USA). Differences were considered significant at *p* < 0.05.

## 3. Results

### 3.1. Lung Physiology during EVLP

**Figure 1:**
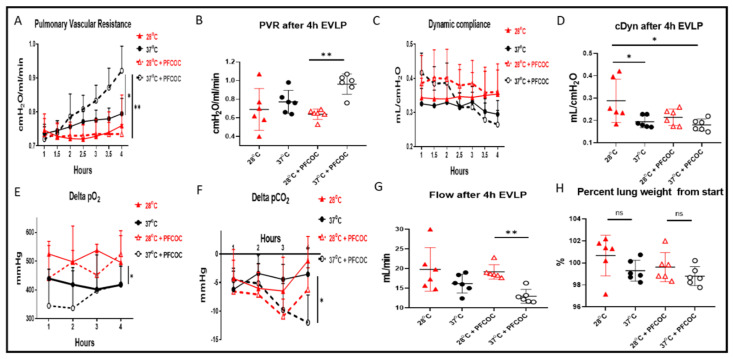
Lung oxygen and carbon dioxide exchange function, PVR, Cdyn and flow during EVLP. (**A**). PVR values during EVLP at 37 °C +/− PFCOC were higher than the subnormothermic conditions but only significantly higher when compared to EVLP at 28 °C with PFCOC (* *p* < 0.05, ** *p* < 0.01). During the 5 min end of EVLP stress test, (**B**) the PVR in the 28 °C + PFCOC EVLP was significantly reduced (** *p* < 0.01) but the similar variations observed at 28 °C without PFCOC were not significant when compared to EVLP at 37 °C without PFCOC. (**C**). Cdyn values during EVLP at 28 °C +/− PFCOC were higher but not significantly higher than the EVLP at 37 °C +/− PFCOC. During the 5 min end of EVLP stress test, (**D**) the Cdyn of the 28 °C without PFCOC was significantly higher than the 37 °C temperature (* *p* < 0.05). (**E**). During EVLP at 28 °C +/− PFCOC, the lung oxygen exchange function was higher when compared to the 37 °C +/− PFCOC conditions (* *p* < 0.05). (**F**). During EVLP at 37 °C + PFCOC condition the lung carbon dioxide exchange function were significantly higher as compared to the 37 °C without PFCOC condition (* *p* < 0.05). (**G**). During the 5 min end of EVLP stress test, in the 28 °C + PFCOC EVLP, the flow was significantly higher (** *p* < 0.01) compared to EVLP at 37 °C with PFCOC. (**H**). The lungs gained weight after EVLP at 28 °C +/− PFCOC but weight gains were not significantly higher when compared to the 37 °C +/− PFCOC conditions.

At 28 °C with PFCOC, we noticed a significantly lower lung PVR when compared to the 37 °C with PFCOC condition (Figure 1A, ** *p* < 0.01) whereas the PVR at 37 °C without PFCOC was significantly lower when compared to the 37 °C with PFCOC condition (Figure 1A, * *p* < 0.05). After the 4-h time subnormothermic EVLP and following the 5 min stress-test, we recorded a significantly lower PVR at 28 °C with PFCOC (Figure 1B, ** *p* < 0.01) compared to the normothermic EVLP with PFCOC. In EVLP done with or without PFCOC at 28 °C we observed higher but not significantly different Cdyn in comparison to the EVLP done with or without PFCOC at 37 °C (Figure 1C). After the 4 h subnormothermic EVLP with PFCOC and following the 5 min stress-test, the Cdyn of the 28 °C EVLP were significantly higher than in the 37 °C temperature with or without PFCOC (Figure 1D, * *p* < 0.05). During the 28 °C EVLP without PFCOC we recorded significantly higher lung oxygen exchange function in comparison to the 37 °C EVLP conditions (Figure 1E, * *p* < 0.05) and significantly higher lung carbon dioxide exchange function in the 37 °C EVLP with PFCOC as compared to the 37 °C without PFCOC condition (Figure 1F, * *p* < 0.05). After the 4 h time subnormothermic EVLP and following the 5 min stress-test, we recorded a significant increase in flow since the 28 °C EVLP with PFCOC were significantly higher than in the 37 °C temperature with PFCOC (Figure 1G, * *p* < 0.01). We also recorded lung percentage weight gain after the 4-h EVLP at 28 °C with or without PFCOC but weight gains were not significantly different from the weight gains observed at 37 °C with or without PFCOC (Figure 1H).

### 3.2. Perfusate Clinical Biochemistry, ATP and MPO Tissue Levels

**Figure 2:**
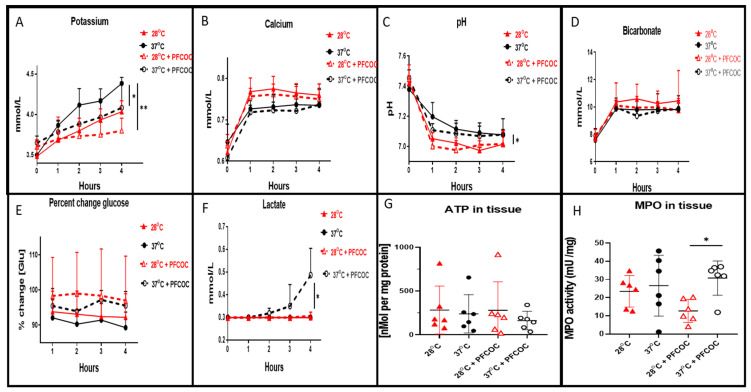
Perfusate clinical biochemistry, ATP and MPO tissue levels. (**A**). During EVLP at 37 °C we recorded significantly increased potassium levels in perfusates when compared to EVLP at 28 °C (* *p* < 0.05) and also potassium at 37 °C was increased in comparison to the perfusates of EVLP at 28 °C with PFCOC (** *p* < 0.01). (**B**). Calcium level in EVLP perfusates at 37 °C +/− PFCOC were lower but were not statistically lower when compared to calcium perfusate levels after EVLP done at 28 °C +/− PFCOC. (**C**). The pH recorded in the 28 °C +/− PFCOC EVLP perfusates were significantly lower than the 37 °C +/− PFCOC (* *p* < 0.05). (**D**). Levels of bicarbonate were lower at 37 °C +/− PFCOC EVLP although not significantly lower than the 28 °C +/− PFCOC EVLP. (**E**). Percent change of glucose from baseline showed higher levels remaining in the perfusates during the EVLP done with PFCOC at 28 °C or 37 °C. (**F**). Lactate levels in the 37 °C + PFCOC EVLP perfusates were significantly increased versus all the other conditions (* *p* < 0.05). (**G**). ATP content of lung tissues after the 28 °C EVLP with or without PFCOC were higher but not significantly different from the lung tissues after the 37 °C EVLP with or without PFCOC conditions. (**H**). MPO lung tissue activities were significantly increased in the 37 °C with PFCOC group when compared to the 28 °C with PFCOC group (* *p* < 0.05).

Perfusate concentrations of potassium during the 4 h EVLP at 28 °C without PFCOC were significantly reduced when compared to 37 °C without PFCOC (Figure 2A, * *p* < 0.05). At 28 °C with PFCOC, potassium levels were also significantly reduced when compared to 37 °C (Figure 2A, ** *p* < 0.01). Perfusate calcium concentrations at 28 °C with or without PFCOC were higher but not statistically different when compared to their respective 37 °C groups (Figure 2B). In Figure 2C, the pH values for the subnormothermic EVLP with or without PFCOC were significantly lower from the pH values of the normothermic conditions (Figure 2C, * *p* < 0.05). The bicarbonate levels measured exiting the lungs were higher at 28 °C without PFCOC but not significantly more over time than the all the other conditions (Figure 2D). The percent change of glucose from baseline value showed higher levels of glucose present in the perfusate at 28 °C or 37 °C with PFCOC versus their respective perfusate at 28 °C or 37 °C without PFCOC (Figure 2E). The normothermic with PFCOC perfusate lactate levels were significantly higher in comparison to all the other conditions (Figure 2F, * *p* < 0.05). After subnormothermic EVLP, the ATP content measured in lung tissue with or without PFCOC were higher but not significantly different with from the normothermic with or without PFCOC conditions (Figure 2G). The MPO tissue activity were higher in the with or without PFCOC 37 °C conditions versus their respective 28 °C conditions nonetheless it was only in the 37 °C with PFCOC condition that this difference was significantly higher when compared to the 28 °C with PFCOC (Figure 2H, * *p* < 0.05).

### 3.3. Cytokines, Chemokines and Mediators of Wound Healing and Tissue Repair in the Perfusate

In Table 1, after the 4 h EVLP time, we report the perfusate cytokines, chemokines and mediators of wound healing and tissue repair levels. The pro-inflammatory mediator TNFα and the pro-inflammatory cytokines IL-6 and IL-7 were significantly lower in the perfusates at 28 °C without (** *p* < 0.01) or with PFCOC (** *p* < 0.01) when compared to the respective 37 °C without or with PFCOC conditions. At 28 °C or 37 °C perfusion without PFCOC and, in comparison to the respective temperature with PFCOC, some chemokines mediators of leukocytes trafficking were significantly lower such as MIP-3α (respectively ** *p* < 0.01 and ** *p* < 0.01), MIP-1α (respectively ** *p* < 0.01 and ns), MCP-1 (respectively ** *p* < 0.01 and ** *p* < 0.01) and GRO/KC (respectively ** *p* < 0.01 and ** *p* < 0.01). Also lower at 28 °C without or with PFCOC were some growth factors such as GM-CSF (respectively * *p* < 0.05 and ** *p* < 0.01), G-CSF (respectively * *p* < 0.05 and ** *p* < 0.01). For the anti-inflammatory cytokines at 37 °C without or with PFCOC, a significantly higher amount as compared to their respective 28 °C without or with PFCOC was recorded for IL-4 (respectively * *p* < 0.05 and * *p* < 0.05) whereas IL-10 was significantly higher only at 37 °C versus the 28 °C condition (* *p* < 0.05). Some analytes such as RANTES, M-CSF, IL-1α, IL-1β, IL-5, IL-12(p70) and IL-17A were without significant difference between groups. Other analytes such as VEGF, IFNγ and the cytokines IL-2, IL-13, IL-18 were undetectable in any of the conditions (not shown). 

## 4. Discussion

Current lung machine perfusion assessment before transplantation are performed at normothermia but subnormothermic perfusion evaluation are already in standard clinical use for several solid organs [9,14,27,28,29,30]. It has been recently demonstrated that PFCOC are useful in lung surfactant therapy [31] and that the systemic administration of PFCOC may have potent anti-inflammatory effects in vivo [19,20,21], ex vivo [22], and in vitro [23,24]. Recently, we also demonstrated with a lung transplantation large animal model the benefits of using PFCOC with normothermic EVLP [22]. Here, we hypothesized that physiological, biochemical and inflammatory parameters in donor grafts may be positively impacted by the simultaneous use of PFCOC and subnormothermia. We monitored donor lung quality during EVLP and assessed airway pressure, pulmonary artery pressure, and oxygen concentration. In the subnormothermic EVLP conditions with or without PFCOC, the lung gas exchange functions for oxygen and carbon dioxide were significantly improved in comparison to the PFCOC normothermic EVLP condition. As would apply here for the 28 °C versus the 37 °C temperature, an obvious explanation is that gas solubility typically decreases as temperature increases. Actually, the two PFCOC groups were transporting oxygen and at the same time acted at better reduce the circulating levels of CO_2_ in the perfusate and the pCO_2_ levels were significantly decreased at 37 °C with PFCOC versus the 37 °C without PFCOC. Consequently, those observations may favor decreased requirement for minute ventilation during EVLP and potentially better protect the injured lungs. Moreover, the lung PVR recorded at 28 °C with PFCOC was significantly lower when compared to the 37 °C with PFCOC condition. Lower temperature in this setting may avoid evaporation of PFCOC due to the Ostwald effect [32]. We also noted with or without PFCOC a higher but not significantly different Cdyn in the 28 °C condition in comparison to the 37 °C EVLP. Following a stress-test performed for 5 min at the end of the 28 °C + PFCOC EVLP we recorded a significant higher flow, a significantly lower PVR and higher Cdyn versus the 37 °C EVLP with PFCOC and similar trends were observed when the 28 °C and 37 °C without PFCOC conditions were compared. The cold perfusate may have had deleterious effect in this setting on the surfactant biophysical properties (surface tension) of the sensitive lung parenchyma and on the blood vessels (vasoconstriction). Nonetheless, the physiological data (PVR, Cdyn, ΔpO_2_) of the subnormothermic with PFCOC condition point towards a non-deleterious and protective effect ending in a better physiological state of the donor graft after the 4 h time of EVLP compared to the 37 °C with PFCOC situation. Our EVLP setting was only for a 4-h time and did probably not trigger any lung tissue remodeling but it has been reported during human whole-body hypothermia that surfactant compositional/structural changes may quickly occurs [33]. Moreover, lung tissue remodeling [34,35,36] and lung surfactant composition are adapted to the cold [37] but only during the long hibernation time, which is a natural and complex physiological and metabolic response to cold temperature developed in mammals and reptiles. Liver and kidneys are both absent of the acellular lung machine perfusion protocols. As a direct consequence of this, the circulating concentration of potassium and lactate are usually increased over the time but here with subnormothermic EVLP we additionally observed that calcium ions were higher and the glucose was less consumed when compared to the normothermic conditions. We did not record significantly different values between the two perfusion temperature conditions for sodium and chloride (data not shown). The cell integrity [38] and the cell death [39] are characterised by changed in physiological levels of potassium. In our setting the potassium concentrations were always lower during the 28 °C EVLP with or without PFCOC. Interestingly, the pH was lower in EVLP done at 28 °C with or without PFCOC whereas bicarbonate concentration at 28 °C EVLP were higher. This reduced pH during subnormothermic EVLP was recorded with a significantly higher lactate concentration in the 37 °C with PFCOC condition. As soon as the normal pathway of glycolysis is routed to produce lactate during anaerobic glycolysis, the metabolic waste product lactate will accumulate over time [40]. Anaerobic metabolism is characterized by lactic acidosis resulting from the excess formation of lactate in the absence of oxygen. For patients undergoing urgent heart transplant on short-term mechanical circulatory support, it has been reported that the preoperative serum lactate levels are a strong independent predictor of worse outcomes [41], but the outcomes for patients who underwent lung transplantation after EVLP remained good despite increased lactate during EVLP [40]. An EVLP setting is characterised by a lack of compensatory mechanisms (i.e., from kidneys or liver) and decreased lactate levels or reduced glucose consumption may indicate aerobic glycolysis in the two 28 °C with or without PFCOC groups. Moreover, and contrasting with changes of lactate or glucose levels attributed to a whole animal metabolism, the changes observed during EVLP results only from the pneumocytes metabolism and occurs at times when energy is required in the absence of oxygen. With subnormothermic EVLP, the improved levels of potassium, lactate and glucose in perfusate point towards a beneficial effect of subnormothermia compared to the 37 °C groups. The presence of PFCOC during subnormothermic lung machine perfusion improved several biochemical parameters of the lung tissue. A sequence of events such as the blood flow loss, a time of cold exposure and ischemia, and a time of reperfusion result in the so-called I/R lesions [42]. MPO enzymes are pro-inflammatory biomarkers present in neutrophilic granulocytes and are released into the extracellular fluid following an inflammatory response or an oxidative stress. MPO activity, released from neutrophilic granulocytes, was significantly lower under subnormothermic PFCOC condition but only lowered in the subnormothermic condition without PFCOC. 

Recently, Gloria et al. [43] and our group [44,45] presented evidences that rat lung grafts treated either with normothermic EVLP or without EVLP have a worst outcome than lung grafts treated before transplantation with subnormothermic EVLP temperatures. In this article, at 28 °C with or without PFCOC, we recorded a significantly lower amount of a selection of pro-inflammatory mediators (i.e., TNFα, IL-6, IL-7) and of chemokines (MIP-3α, MIP-1α, MCP-1, GRO/KC) in the perfusates. Interestingly, some anti-inflammatory cytokines such as IL-10 and IL-4 were also low in the subnormothermic conditions. At both 28 °C and 37 °C the pro- or anti-inflammatory mediators levels were mostly higher in the absence of PFCOC. This phenomenon is in accordance with the already reported properties of PFCOC to reduce the inflammatory response both in vivo [19,20,21], ex vivo [22] and in vitro [23,24].

This study has some limitations. First, lung function was assessed during EVLP but we did not organize a post-EVLP transplantation of the lungs and survival studies into recipient animals. Second, during EVLP, the release of pro-inflammatory cytokines such as IL-6 in the perfusate may have been attenuated by the use of a leukocyte filter. As reported by others [46], such an absence of a leukocyte filter may have impaired the quality of all the lung grafts. This is the first study with subnormothermic EVLP and PFCOC, in a clinically relevant rat donor lung model, that present strong beneficial potential evidenced by improved physiological parameters and attenuation of I/R injury when compared to the normothermic temperature. We showed that the 28 °C EVLP has significantly reduced pro-inflammatory cytokine responses and also pCO_2_ levels decreased in the perfusates, that is even enhanced with the presence of PFCOC when compared to normothermic temperature. Taking in account all results, this study suggests that 28 °C EVLP with PFCOC is a non-inferior setting in comparison to the clinically approved normothermic setting and would be appropriate to reduce I/R injury. We plan a well-designed large-animal study with the hypothesis that subnormothermic EVLP and PFCOC will better predict human trial outcomes. 

## Figures and Tables

**Table 1:** Perfusate cytokines, chemokines and mediators of wound healing and tissue repair in the study groups.

Analytes ^1^	Control	Control	PFCOC	PFCOC
	Normo. (*n* = 6)	Subnormo. (*n* = 6)	Normo. (*n* = 6)	Subnormo. (*n* = 6)
TNF-α	1345 (1148)	53.9 (78.2) **	1197 (924.7)	34.4 (26.1) **
MCP-1	100.7 (40.75)	22.85 (10.05) **	97.95 (46.43)	19.71 (8.89) **
GM-CSF	1.09 (0.70)	0.52 (0.16) *	0.51 (0.12)	0.45 (0.01) **
RANTES	11.67 (5.38)	6.80 (3.37)	19.16 (7.97)	15.61 (5.09)
MIP-3α	7.88 (8.36)	0.58 (0.03) **	8.15 (6.36)	0.5 (0.01)**
MIP-1α	2184 (1321)	319 (119.8) **	1846 (1232)	950 (647.1)
M-CSF	0.58 (0.41)	0.69 (0.61)	0.64 (0.62)	0.53 (0.58)
G-CSF	0.14 (0.15)	0.04 (0.004) *	0.07 (0.09)	0.03 (0.001) **
GRO/KC	4096 (2734)	204.7 (242) **	3831 (2350)	288 (198.7) **
IL-1α	0.09 (0.001)	0.09 (0.003)	0.49 (0.63)	0.49 (0.37)
IL-1β	8.16 (5.54)	10.04 (4.14)	11.45 (5.07)	11.53 (3.01)
IL-4	1.16 (0.84)	0.41 (0.12) *	0.79 (0.82)	0.61 (0.38) *
IL-5	5.35 (6.05)	3.95 (2.33)	3.85 (2.15)	3.35 (2.46)
IL-6	143.8 (86.42)	3.03 (0.02) **	137.4 (76.89)	2.97 (0.009) **
IL-7	0.22 (0.05)	0.20 (0.003) **	0.33 (0.34)	0.18 (0.001) **
IL-10	3.473 (2.98)	0.51 (0.63) *	3.40 (4.34)	4.23 (8.66)
IL-12(p70)	1.38 (1.08)	0.76 (1.05)	0.62 (0.85)	0.51 (0.72)
IL-17A	0.96 (0.25)	0.8 (0.0)	0.98 (0.44)	0.8 (0.0)

^1^ Cytokines, chemokines and mediators of wound healing and tissue repair in (pg/mL). Summary of the means (M) and the standard deviations (SD) from the perfusates after 4 h of EVLP with or without PFCOC. Note * *p* ≤ 0.05; ** *p* ≤ 0.01; Normo, normothermic; Subnormo, subnormothermic.

## Data Availability

The data sets generated during and/or analyzed during the current study are available from the corresponding author on reasonable request.

## Abbreviations

Cdyn	dynamic compliance
EVLP	ex vivo lung perfusion
FiO_2_	inspired oxygen fraction
I/R	ischemia-reperfusion
MPO	myeloperoxidase
PAP	pulmonary arterial pressure
PEEP	positive end-expiratory pressure
PFCOC	perfluorocarbon-based oxygen carriers
PVR	pulmonary vascular resistance
ROS	reactive oxygen species
